# Chronic unpredictable mild stress produces depressive-like behavior, hypercortisolemia, and metabolic dysfunction in adolescent cynomolgus monkeys

**DOI:** 10.1038/s41398-020-01132-6

**Published:** 2021-01-04

**Authors:** Teng Teng, Carol A. Shively, Xuemei Li, Xiaofeng Jiang, Gretchen N. Neigh, Bangmin Yin, Yuqing Zhang, Li Fan, Yajie Xiang, Mingyang Wang, Xueer Liu, Mengchang Qin, Xinyu Zhou, Peng Xie

**Affiliations:** 1 Department of Neurology, The First Affiliated Hospital of Chongqing Medical University, Chongqing, China; 2 NHC Key Laboratory of Diagnosis and Treatment on Brain Functional Diseases, The First Affiliated Hospital of Chongqing Medical University, Chongqing, China; 3 Section of Comparative Medicine, Department of Pathology, Wake Forest School of Medicine, Winston-Salem, NC 27101 USA; 4 Department of Psychiatry, Shaoxing Seventh People’s Hospital, Shaoxing, China; 5 Departments of Anatomy and Neurobiology, Virginia Commonwealth University, Richmond, VA 23284 USA; 6 Department of Neurology, The Second Affiliated Hospital of Chongqing Medical University, Chongqing, China; 7 Department of Psychiatry, The First Affiliated Hospital of Chongqing Medical University, Chongqing, China

**Keywords:** Depression, Neuroscience

## Abstract

Adolescent depression is a common and serious mental disorder with unique characteristics that are distinct from adult depression. The adult non-human primate stress-induced model of depressive-like behavior is an excellent model for the study of mechanisms; however, an adolescent nonhuman primate model is still lacking. Ten male adolescent cynomolgus monkeys were divided into a chronic unpredictable mild stress (CUMS, *n* = 5) group and a control (CON, *n* = 5) group by age and weight-matched pairs. The CUMS group was exposed to multiple unpredictable mild stressors for five cycles over 55 days. At baseline, there were no differences between CUMS and CON groups. At endpoint, the CUMS group demonstrated significantly higher depressive-like behavior (huddle posture), and significantly lower locomotion compared with the CON group. Furthermore, depressive-like behavior increased from baseline to endpoint in the CUMS group, but not changed in the CON group. In the attempt for apple test, the CUMS group made significantly fewer attempts for the apple than the CON group. In the human intruder test, the CUMS group showed significantly higher anxiety-like behaviors in the stare phase than the CON group. Hair cortisol level was significantly higher in the CUMS group than the CON group at endpoint, and was also elevated from baseline to endpoint. Metabolic profiling of plasma at endpoint identified alterations in metabolite pathways which overlapped with those of adolescent depression patients. CUMS can induce depressive-like and anxiety-like behaviors, hypercortisolemia, and metabolic perturbations in adolescent cynomolgus monkeys. This is a promising model to study the mechanisms underlying adolescent depression.

## Introduction

Major depressive disorder (MDD) in adolescents is a growing public health concern worldwide, with an estimated point prevalence of about 7.1%^1^. The course of MDD in adolescents is often characterized by undifferentiated symptoms, frequent recurrence, protracted episodes and comorbid psychiatric disorders^2^. The first episode of depression commonly starts in adolescence, and earlier onset is related to greater risk for recurrence^3^. Compared with adults, youth with depression experience more serious impairments in global functioning, an increased risk of tobacco smoking and other substance abuse^2^. Moreover, suicide is the third leading cause of death in adolescents; and among depressed youth, 29% experience suicidal thoughts and 11% attempt suicide^4^. However, the underlying molecular mechanisms of MDD in adolescents appear complex and are poorly understood. Several studies showed that the pathophysiology, neurobiological mechanisms, and treatment response of adolescent depression diverges from adult depression^5,6^.

Animal models have played an essential role in exploring the underlying molecular mechanisms of depression and testing the efficacy of pharmacotherapeutic interventions^7^. Rodent models are commonly used in assessments of depressive-like behaviors; however, the non-human primate (NHP) stress-induced model of depressive-like behaviors offers an excellent model to study mechanisms due to the similarity to humans in the structure and function of the central nervous system, neurodevelopment, social behavior, emotion regulation and stress response^8^. Our previous studies have established two models of depressive-like behavior in adult monkey, a naturally occurring model of depressive-like behavior and a social plus visual isolation-induced model of depressive-like behavior^9,10^. Unfortunately, these models do not provide insight to the underlying molecular mechanisms of depression in adolescents. Naturally occurring depression has not been reported in subadult monkeys. Early life stress (e.g., physical or sexual abuse, trauma) is a risk factor for adolescent depression in humans^11^. Therefore, we adapted a chronic unpredictable mild stress (CUMS) paradigm used in rodent models of depression^12^ to induce depressive-like behavior in adolescent cynomolgus monkeys.

## Materials and methods

### Subjects

Ten male adolescent cynomolgus monkeys (21–52 months old) were selected and divided into two groups with age and weight-matched pairs: a chronic unpredictable mild stress group (CUMS, *n* = 5) and a control group (CON, *n* = 5). All the selected subjects were reared in large stable social groups for the first year of their life. Age and body weight of the subjects at baseline are shown in Supplementary Table S1. The living environment and animal care procedures were detailed in our previous reports^9,10^. Briefly, all subjects were housed in single cages (1.0 m × 1.5 m × 1.5 m, L × W × H) under a 12 h light/dark cycle (≥18 °C, 40–70% relative humidity), provided water ad libitum, and fed fresh fruits, vegetables and monkey chow twice daily. The CUMS group and CON group were housed in two separate rooms. All husbandry and veterinary care was provided by the cynomolgus monkey breeding base (Zhongke Experimental Animal Co., Ltd., located in Xishan Island, Suzhou, China). Animals were maintained under an experiment protocol approved by the Ethics Committee of Chongqing Medical University (approval no.: 20180705) in accordance with the recommendations of ‘The use of non-human primates in research’^13^ and ‘Guide for the Care and Use of Laboratory Animals’^14^. We also performed matched pairs design to minimize the number of subjects, while maintaining statistical power following the principle of NC3Rs (National Centre for the Replacement, Reduction and Refinement, https://www.nc3rs.org.uk/).

### Experiment procedures

**Fig. 1:**
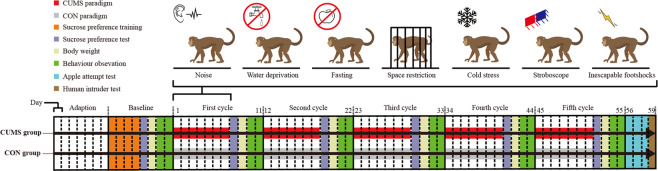
Overview of the experiment protocol. CUMS paradigm (red) and CON paradigm (gray) were conducted to each group, respectively.

Before starting the experiment, the ten subjects were adapted to the housing conditions for seven days, and then baseline tests were conducted for eight days. The experimental phase was 55 days in length. The CUMS group was exposed to seven days of chronic unpredictable mild stressors followed by a four-day behavior observation period; this regimen was conducted for five cycles. Stressors were modified from our previous rodent studies^15–18^ and included noise (100 db, 12 h), water deprivation (12 h), fasting (24 h), space restriction (4 h), cold stress (10 °C, 10 min), exposure to stroboscope (12 h) and inescapable footshocks (6 V, 10–15 s, 3–4 times). We used two different stressors in one day, and the same stressor was not scheduled in two adjacent days. The CON group was handled as usual daily throughout the experiment. The details of the stressors and special caring for the monkeys during the interval of stressors are provided in the Supplementary Table S2. The overall schedule of the experiment is shown in Fig. 1.

### Behavioral observations and tests

Cynomolgus monkeys’ behaviors were videotaped for one hour between 8:00 and 10:00 a.m., respectively at baseline and the end of each stress cycle. The SONY HD cameras were fixed outside the cage with no blind point of the monkeys and all personnel stayed away from the room to avoid disturbing the monkeys. Based on our previous Macaca fascicularis ethogram development^19^, we defined nine behaviors of cynomolgus monkeys in a single-cage environment: huddle posture, locomotion, stereotyped behaviors, response to environment, climbing, self grooming, playing with the ball, observation, and sitting. The definitions and pictures of these behaviors for this study are shown in Supplementary Table S3. Huddle posture (self-clasping with head at or below the shoulders during the waking state) was considered as the core behavior representing depressed mood in cynomolgus monkeys^7^. Decreased time spent in locomotion (walking in the cage) was also reported in depressed macaques in previous studies^7,20,21^.

We measured body weight and sucrose preference test (SPT)^22^ at baseline and the end of each stress cycle. In addition, the attempt for apple test (AAT)^23^ and human intruder test (HIT)^24^ were conducted at the end of the five stress cycles. In the AAT, the fewer attempts for apples than controls was considered as anhedonia, which is a core symptom of patients with depression^23^. In the HIT, the total time spent freezing, scratching, yawning, and showing a fear grimace were defined as anxious behaviors^24^. In the SPT, monkeys were exposed to pure water or 5% sucrose water for one hour after 12 h water-deprivation. The detailed procedures of the above tests are shown in Supplementary Table S4, and definitions and pictures of behaviors in the AAT, HIT and SPT are shown in Supplementary Table S5.

### Measurement of hair and blood cortisol

At the baseline and the end of five stress cycles, hair and blood samples of ten subjects were collected at 8:00–9:00 a.m. before feedings. Hair from posterior vertex region of scalp was clipped without anesthetic by using a pair of scissors and stored in a small aluminum foil pouch^25^. Blood was collected from the femoral vein into 5 mL vacutainer tubes containing heparin lithium. All blood samples were centrifuged at 1500 × *g* for 15 min, divided into 150 μL plasma aliquots, and stored at −80 °C. The method of hair cortisol extraction was described in detail in a previous study^26^. Briefly, hair samples were washed in isopropanol twice (3 min each) and dried at 35 °C (8 h) to remove surface contaminants. Then, samples were pulverized using grinding miller. Powdered hair (500 mg) was weighed and incubated in 10 mL of methanol to extract cortisol with a slow rotation under room temperature for 24 h. The samples were then centrifuged at 10,000×*g* for 5 min, and 5 mL of the supernatant was pipetted into a centrifuge tube and dried with nitrogen gas. Finally, the precipitated extract was reconstituted with 0.5 mL of PBS and stored at −20 °C until assayed. The cortisol concentrations of hair and blood were quantified by radio immunoassays (RIAs) by using a commercially available kit (Monkey Cortisol RIA JL21813, China) in a double-blind procedure. Each sample was tested three times and the mean of the three cortisol values was used for statistical analysis.

### Metabolomic analysis of plasma samples

The procedures for sample preparation and liquid chromatography-mass spectrometry (LC-MS) analysis have been published previously^6^. Briefly, at the end of five stress cycles, blood sample of ten subjects were collected at 8:00–9:00 a.m. before feedings. For LC-MS analysis, the samples were re-dissolved in acetonitrile/water solvent. Analyses were performed using an ultra-high performance liquid chromatography (1290 Infinity LC, Agilent Technologies) coupled to a quadrupole time-of-flight (AB SciexTripleTOF 6600). For the hydrophilic interaction liquid chromatography separation, samples were analyzed using a 2.1 mm × 100 mm ACQUIY UPLC BEH 1.7 µm column (waters, Ireland). The raw MS data (wiff.scan files) were converted to MzXML files. For the multivariate statistical analysis, the soft independent modeling of class analogy (SIMCA) software (version 14.0, Umea, Sweden) was used. Principal components analysis (PCA) was used to observe the distributions of all samples and obtain a general overview of metabolic pattern changes. Orthogonal partial least squares-discriminate analysis (OPLS-DA) was used to find variables associated to the separation and calculate the variable influence on projection (VIP). The metabolites with VIP > 1 and *P*-values <0.05 (Wilcoxon Signed Rank Test) were defined as significantly different metabolites. Then, we compared the metabolic phenotypes of adolescent CUMS monkeys with adolescent MDD patients.

### Statistical analysis

Statistical analyzes were performed by using IBM SPSS Statistics for Windows, Version 25.0 (IBM Corp., Armonk, NY, USA). We matched pairs with age and weight between CUMS group and CON group. The results of behaviors and cortisol levels were expressed as the mean ± SEM. Wilcoxon Signed Rank Test was used for all statistical analysis. *P*-values less than 0.05 were considered statistically significant. Pearson’s correlation was used to calculate the relationship between hair and plasma cortisol levels.

## Results

### Behavior changes between CUMS group and CON group

**Fig. 2:**
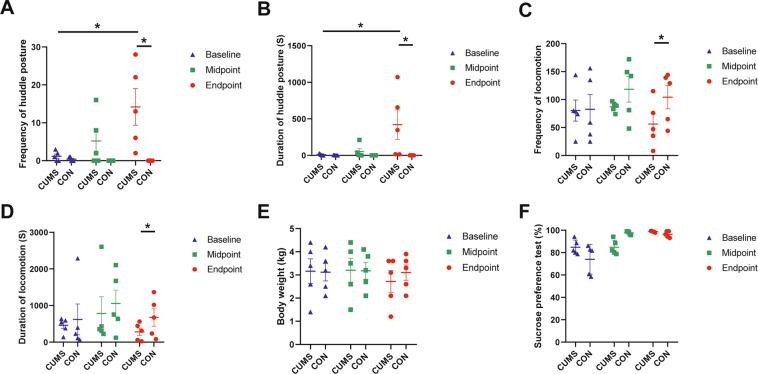
The results of the huddle posture, locomotion, body weight and sucrose preference test in CUMS and CON groups at baseline, midpoint (after three stress cycles) and endpoint (after five stress cycles). **A** The frequency of huddle posture. **B** The duration of huddle posture. **C** The frequency of locomotion. **D** The duration of huddle locomotion. **E** Body weight. **F** Sucrose preference test. *P < 0.05.

The results of huddle posture, locomotion, SPT, and body weight are shown in Fig. 2 and the detail of all the observed behaviors in Supplementary Table S6. At baseline and at the end of three stress cycles (midpoint), there were no significant differences in behavior, body weight, or the SPT between CUMS group and CON group. At the end of five stress cycles (endpoint), compared with the CON group, the CUMS group demonstrated a significantly higher frequency (Z = −2.0226, *P* = 0.0431; Fig. 2A) and longer duration of huddle posture (*Z* = −2.0226, *P* = 0.0431; Fig. 2B); and showed a significantly lower frequency (*Z* = −2.0226, *P* = 0.0431; Fig. 2C) and shorter duration of locomotion (Z = −2.0226, *P* = 0.0431; Fig. 2D). However, there were no significant differences of body weight (*Z* = −1.8257, *P* = 0.0679; Fig. 2E), SPT (*Z* = −1.4832, *P* = 0.1380; Fig. 2F) and other defined behaviors (Supplementary Table S6) between CUMS and CON groups at endpoint. In addition, the frequency (*Z* = −2.0226, *P* = 0.0431; Fig. 2A) and duration (*Z* = −2.0226, *P* = 0.0431; Fig. 2B) of huddle posture in the CUMS group was significantly increased from baseline to endpoint, while these metrics did not change in CON group (*Z* = −1.4142, *P* = 0.1573; Fig. 2A, B).

**Fig. 3:**
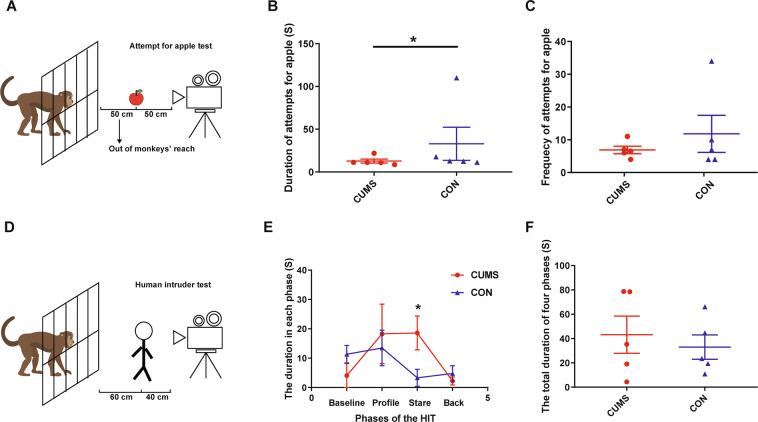
The results of attempt for apple test (AAT) and human intruder test (HIT) in CUMS and CON groups. **A** The schematic of the AAT. **B** The duration of attempts for apple for total three days. **C** The frequency of attempts for apple for total three days. **D** The schematic of the HIT. **E** The duration of the anxiety-like behaviors in each phase. **F** The total duration of the anxiety-like behaviors in four phases. *P < 0.05. Subject S2 in CUMS group dropped out of AAT and HIT due to diarrhea, so we imputed the median value for each behavior in CUMS group as the value of S2 in statistical analysis.

The results of the AAT and HIT are shown in Fig. 3 (detailed in Supplementary Table S7). In the AAT (Fig. 3A), compared with the CON group, those in the CUMS group had significantly shorter durations of attempts for the apple than the CON group (*Z* = −2.0226, *P* = 0.0431; Fig. 3B); there was no difference between groups for the frequency of attempts for the apple (*Z* = −0.6742, *P* = 0.5002; Fig. 3C). In the HIT (Fig. 3D), compared with CON group, the CUMS group had significantly longer duration of anxiety-like behaviors in the stare phase (Z = −2.0226, *P* = 0.0431; *P* = 0.0431, Fig. 3E), but not in the other three phases (*P*-value from 0.1088 to 0.6858; Fig. 3E) or the total duration of all four phases (*Z* = −0.4045, *P* = 0.6858; Fig. 3F).

### Cortisol levels in hair and plasma samples

**Fig. 4:**
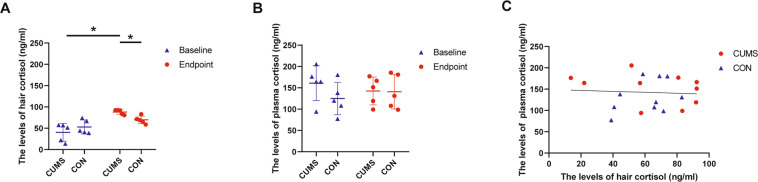
The level of cortisol. The level of hair (**A**) and plasma (**B**) cortisol in CUMS and CON groups at baseline and endpoint (after five stress cycles); the correlationship between hair and plasma cortisol level (**C**). *P < 0.05.

Cortisol levels in hair and plasma samples are shown in Fig. 4 (detailed in Supplementary Table S8). Hair (*Z* = −1.2136, *P* = 0.2249; Fig. 4A) and plasma (*Z* = −0.4045, *P* = 0.6858; Fig. 4B) cortisol levels were not different between CUMS and CON groups at baseline. Hair cortisol levels were significantly higher in the CUMS group compared with the CON group at endpoint (*Z* = −2.0226, *P* = 0.0431; Fig. 4A), but there was no significant difference in plasma cortisol levels (*Z* = −0.1348, *P* = 0.8927; Fig. 4B). In addition, hair cortisol levels in the CUMS group rose significantly from baseline to endpoint (*Z* = −2.0226, *P* = 0.0431; Fig. 4A), but not in the CON group (*Z* = −1.2136, *P* = 0.2249; Fig. 4A). Plasma cortisol levels between baseline and endpoint did not significantly change in either group (CUMS: *Z* = −0.9439, *P* = 0.3452; CON: *Z* = −0.9439, *P* = 0.3452; Fig. 4B). Moreover, there was no significant correlation between the cortisol values measured in hair and plasma (*r* = −0.07, *P* = 0.7835; Fig. 4C).

### Metabolomic analysis of plasma

**Fig. 5:**
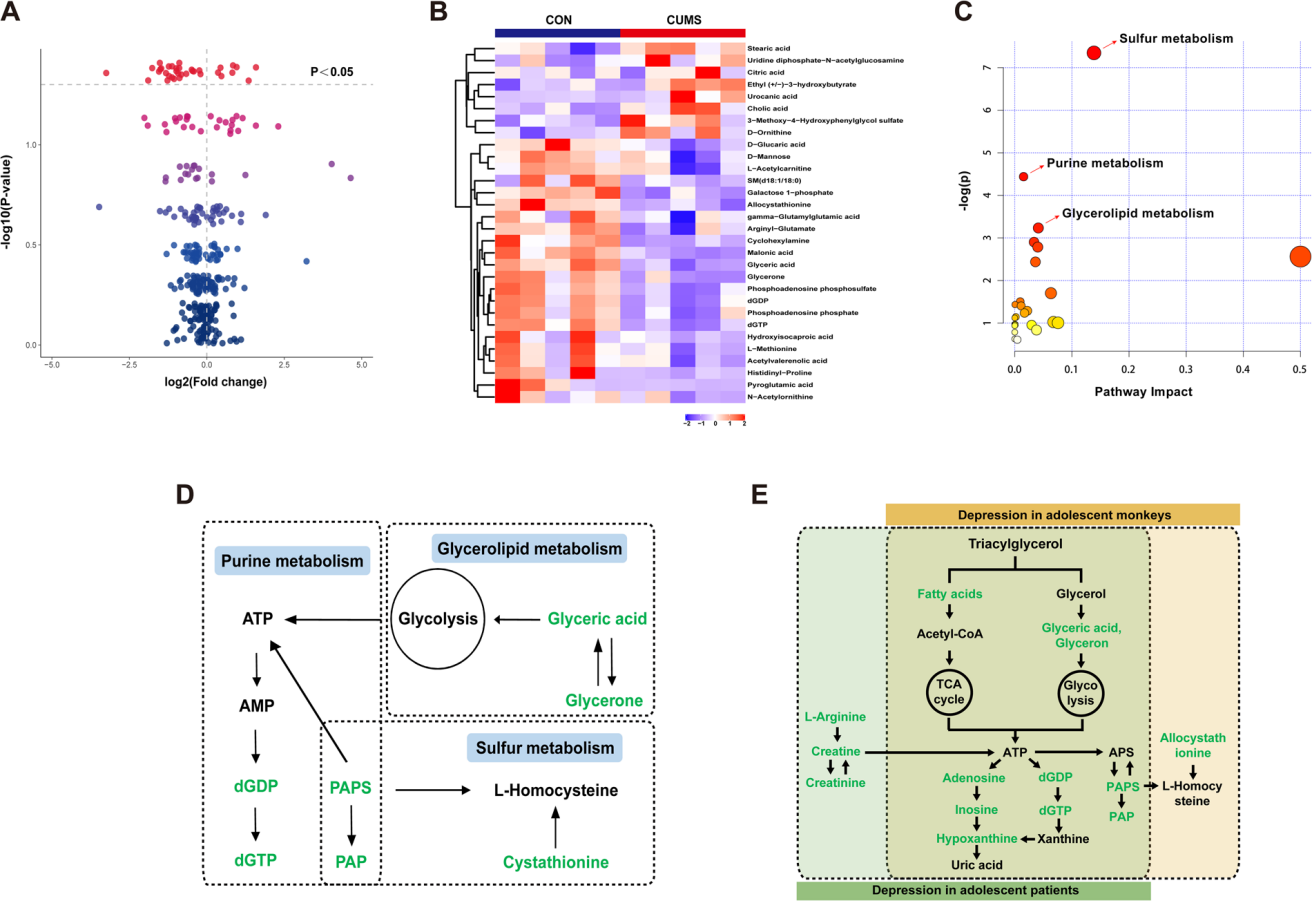
Plasma metabolomics analysis in CUMS and CON groups. **A** The volcano plot for the differential metabolites in CUMS and CON groups; red nodes = *P* < 0.05. **B** The heat plot for the differential metabolites in CUMS and CON groups. **C** The pathway analysis of differential metabolites of CUMS and CON groups in MetaboAnalyst; red nodes = *P* < 0.05. **D** A simplified interaction diagram of the altered pathways in CUMS and CON groups. Metabolites in green decreased. **E** A simplified interaction diagram of altered pathways between adolescent depression monkeys and patients. Metabolites in green decreased.

Using LC-MS metabolomic profiling of plasma from CUMS and CON groups, 145 metabolite components were identified in positive ion mode and 158 were identified in negative ion mode. Based on OPLS-DA model (detailed in Supplementary Fig. S1), a total of 30 differential metabolites were identified between the CUMS group and the CON group using the criteria of VIP > 1 and *p* < 0.05 (Supplementary Table S9), including 23 downregulated metabolites and 7 upregulated metabolites. The volcano plot and heat plot for the differential metabolites in CUMS vs. CON are displayed in Fig. 5A, B. MetaboAnalyst 4.0 was applied to reveal the potential biological processes of 30 differential metabolites in CUMS vs. CON. The pathway analysis indicated that there were significant perturbations in sulfur metabolism, purine metabolism, and glycerolipid metabolism pathways in the CUMS group compared to the CON group (Fig. 5C), which can be connected through altered metabolites (Fig. 5D).

We compared our previous metabolomics data of adolescent MDD patients^6^ with the current metabolomic data from adolescent CUMS monkeys. Pathways of adolescent CUMS monkeys that were significantly different from those of the CON group and the same pathways in adolescent MDD patients are shown in Supplementary Table S10. Disturbances of metabolism pathways were combined together in adolescent CUMS monkeys and adolescent MDD patients (Fig. 5E).

## Discussion

In this study, for the first time, exposure of adolescent monkeys to CUMS resulted in depressive-like behavior, including increased huddle posture, decreased locomotion, and anhedonia as evidenced by decreased attempts for the apples. Furthermore, hair cortisol levels were elevated in the CUMS group, and the biomolecular differences in plasma of CUMS monkeys compared with control monkeys were similar to those observed in adolescent depression patients^6^. Thus, both behavioral and biological results suggested that the CUMS paradigm in this study was effective in producing behavioral and physiological changes in adolescent monkeys that are consistent with observations in depressed adolescent humans.

Two core symptoms of patients with MDD are depressed mood and lack of interest or anhedonia^27^. In the rodent model of depressive-like behavior, increased immobility time in the forced swim test is representative of depressive-like behavior, and the decreased preference for sucrose in the SPT is representative of anhedonia^28^. Correspondingly, in the monkey model of depressive-like behavior, increased huddle posture and decreased locomotion are the representative of ‘depressed mood’, but there is no accepted behavioral test for lack of interest or anhedonia in monkeys^7,20,21^. Attempts to validate the sucrose preference test to measure anhedonia have resulted in contradictory results. Less sucrose consumption was observed in adult rhesus macaques following chronic glucocorticoid exposure^29^, a model of depressive-like behavior, while more was observed in adolescent monkeys that demonstrated depressive-like behaviors following social isolation^30^. Recently, Fan et al. reported that the attempt for apple test (AAT) appeared to reliably measure anhedonia in cynomolgus monkeys^23^. In our study, SPT and AAT were both conducted to examine anhedonia, but only the AAT differentiated between the control and stressed groups. It may result from two possible reasons. First, the monkeys are more sensitive to the taste of ethanol in ripe apples than rodents, so apples, with both ethanol and sucrose, are a stronger stimulus than pure sucrose solutions^31^. Second, the examination of anhedonia can work better when increasing the effort of the monkeys to acquire the sucrose in NHP study^32^. In this study, the AAT was a useful test of anhedonia in depressed monkeys.

Anxious behaviors in macaques manifest as an increased rate of self-directed behaviors^33^. The human intruder test (HIT) is widely used as a challenge test to elicit anxiety-like behaviors in laboratory monkeys^24^. The CUMS group exhibited increased self-directed behaviors in the HIT suggesting increased anxiety. Thus, adolescent CUMS monkeys displayed both depressive-like behaviors and anxiety-like behaviors. This is important because anxiety and depression are frequently comorbid in children and adolescents, and these disorders are likely to share some commonalities in pathogenesis^34^.

Dysfunction of the hypothalamic-pituitary-adrenal (HPA) axis is a characteristic of depressive disorders, and hypercortisolemia is a common biological marker of HPA axis dysfunction^35^. In this study, hair cortisol levels of adolescent CUMS monkeys were higher than controls and also elevated from baseline to endpoint, which is consistent with previous studies of depression in non-human primates^36^. However, plasma cortisol levels were not different between the two groups. It may be that plasma cortisol is more reactive to external environmental cues (e.g., sample collection^37^, food intake^38^, and circadian rhythms^39^), while hair cortisol is relatively stable, representing the total activity of HPA axis over the preceding months^40^. Thus, hair hypercortisolemia in the adolescent CUMS monkeys represents a biological characteristic of depression.

We found three altered plasma metabolite pathways between CUMS and CON groups, including purine metabolism, sulfur metabolism and glycerolipid metabolism pathways. All of these three metabolism pathways were reported to be dysregulated in MDD patients^41,42^. Comparing with our previous study of plasma metabolomic from adolescent MDD patients^6^, two of the three altered metabolism pathways overlapped between adolescent monkeys that underwent CUMS and depressed patients. The downstream metabolites of triacylglycerol and glycerolipid metabolism pathway were decreased, and this caused downregulation of metabolic pathways related to energy (tricarboxylic acid cycle and glycolysis), which play crucial roles in the mechanisms of MDD^43^. Moreover, the antidepressant effects for MDD patients of the ω-3 polyunsaturated fatty acids, eicosapentaenoic acid (EPA) and docosahexaenoic acid (DHA) in the triacylglycerol and glycerolipid metabolism pathways have been widely verified^44,45^. In addition, we found that adenosine triphosphate (ATP), which has been confirmed as a key factor involved in the biological mechanisms of MDD^46^, was in the center of all altered metabolites. Our findings suggest that the downregulation of ATP and its related metabolites is associated with alterations following chronic bouts of repeated stressors and the pathology of adolescent depression. However, there were some differences in altered pathways between adolescent monkeys exposed to CUMS and depressed patients, which may result, in part from differences in diet^47^.

There are some limitations in this study. First, according to the requirements of ethics, we chose the smallest sample size. Second, in order to reduce the effect of sex hormones on the experiment, we only selected male adolescent cynomolgus monkeys in this study. However, it is unknown whether these observations will generalize to females. Third, although all procedures followed the experiment protocol were approved by the Ethics Committee, and were in accordance with the international guidelines, increased stress was experienced by the subjects in CUMS group. To meet experimental requirements set by the institutional animal care and use committee, additional care was offered daily after the stressors (e.g., additional fruit and vegetable, social contact, and toys) which may also have influenced the outcomes of the study. Nevertheless, we recognize the important contribution of these subjects to our understanding of depressive-like behaviors in adolescents. Fourth, there are many biological constructs suggested so far in the depression patients. However, in this study, we only tested the current two most recognized biological construct validities by HPA-axis activity and metabolic perturbations in adolescent depression. And, we did not test the predictive validity in this study. Last but not least, etiology of depression is a complex process and one model can only reflect one aspect of the etiology. Thus, chronic unpredictable mild stress may not sufficiently reflect all the causes of adolescent depression.

In summary, we observed that CUMS induced depressive-like behavior, anxiety-like behavior and hypercortisolemia in adolescent male cynomolgus monkeys. The attempt for apple test appeared to be a reliable indicator of anhedonia in cynomolgus monkeys. Metabolic changes in CUMS monkeys were mainly concentrated in pathways related to sulfur metabolism, purine metabolism, and glycerolipid metabolism and these changes were similar to those of adolescent depression patients. These findings suggest that CUMS adolescent monkeys may be a useful model to study the underlying mechanisms of adolescent MDD in humans.

## Supplementary information

Supplementary materials

Table S1

Table S2

Table S3

Table S4

Table S5

Table S6

Table S7

Table S8

Table S9

Table S10

Figure S1
