# Correction to: Chronic unpredictable mild stress produces depressive-like behavior, hypercortisolemia, and metabolic dysfunction in adolescent cynomolgus monkeys

**DOI:** 10.1038/s41398-021-01251-8

**Published:** 2021-02-15

**Authors:** Teng Teng, Carol A. Shively, Xuemei Li, Xiaofeng Jiang, Gretchen N. Neigh, Bangmin Yin, Yuqing Zhang, Li Fan, Yajie Xiang, Mingyang Wang, Xueer Liu, Mengchang Qin, Xinyu Zhou, Peng Xie

**Affiliations:** 1 Department of Neurology, The First Affiliated Hospital of Chongqing Medical University, Chongqing, China; 2 NHC Key Laboratory of Diagnosis and Treatment on Brain Functional Diseases, The First Affiliated Hospital of Chongqing Medical University, Chongqing, China; 3 Section of Comparative Medicine, Department of Pathology, Wake Forest School of Medicine, Winston-Salem, NC 27101 USA; 4 Department of Psychiatry, Shaoxing Seventh People’s Hospital, Shaoxing, China; 5 Departments of Anatomy and Neurobiology, Virginia Commonwealth University, Richmond, VA 23284 USA; 6 Department of Neurology, The Second Affiliated Hospital of Chongqing Medical University, Chongqing, China; 7 Department of Psychiatry, The First Affiliated Hospital of Chongqing Medical University, Chongqing, China

**Keywords:** Depression, Neuroscience

Correction to: *Translational Psychiatry*

10.1038/s41398-020-01132-6 published online 04 January 2021

The original version of this article unfortunately contained a mistake in the Acknowledgement section. The acknowledgement of funding from National Institutes of Health grant number R01HL087103 was incorrect. No funding from the grant was used to support the study. The authors apologize for the mistake. The original article has been corrected.

