# Supplementary material for: Chronic unpredictable mild stress produces depressive-like behavior, hypercortisolemia, and metabolic dysfunction in adolescent cynomolgus monkeys
Source: Transl Psychiatry. 2021 Jan 4;11:9. doi: 10.1038/s41398-020-01132-6 (PMC7791128; doi:10.1038/s41398-020-01132-6)
Supplement: Supplementary file 1 — Supplementary materials [file 41398_2020_1132_MOESM1_ESM.docx]

**Supplementary Materials for:**

**Chronic unpredictable mild stress produces depressive-like behavior, hypercortisolemia, and metabolic dysfunction in adolescent cynomolgus monkeys**

Teng Teng, Carol A. Shively, Xuemei Li, Xiaofeng Jiang, Gretchen N. Neigh, Bangmin Yin, Yuqing Zhang, Li Fan, Yajie Xiang, Mingyang Wang, Xueer Liu, Mengchang Qin, Xinyu Zhou, Peng Xie

The supplementary materials of this article contain Tables S1-S10 and Fig. S1.

**Table S1.** Age and body weight of the subjects at baseline

**Table S2.** The definitions of chronic unpredictable mild stressors

**Table S3.** The definitions and pictures of behavioral observations

**Table S4.** The detailed procedures of attempt for apple test, human intruder test, sucrose preference test

**Table S5.** The definitions and pictures of behavioral tests

**Table S6.** The detailed results of the all the observed behaviors in CUMS (S) and CON (C) groups at baseline, midpoint (after three stress cycles) and endpoint (after five stress cycles)

**Table S7.** The detailed results of the attempt for apple test, and the behaviors in each phase of human intruder test in CUMS (S) and CON (S) groups

**Table S8.** The levels of hair and plasma cortisol in CUMS (S) group and CON (C) group at baseline and endpoint (after five stress cycles)

**Table S9.** Identified differential metabolites in the plasma between the CUMS and CON groups

**Table S10.** The significantly altered pathways of depression in adolescent monkeys and patients

**Fig. S1.** Orthogonal partial least squares-discriminate analysis (OPLS-DA) score plots of CUMS group versus CON group in positive mode (A) and negative mode (B) in adolescent monkeys
