# Supplementary material for: Chronic unpredictable mild stress produces depressive-like behavior, hypercortisolemia, and metabolic dysfunction in adolescent cynomolgus monkeys
Source: Transl Psychiatry. 2021 Jan 4;11:9. doi: 10.1038/s41398-020-01132-6 (PMC7791128; doi:10.1038/s41398-020-01132-6)
Supplement: Supplementary file 2 — Table S1 [file 41398_2020_1132_MOESM2_ESM.docx]

**Table S1.** Age and body weight of the subjects at baseline

|  | C1 | S1 | C2 | S2 | C3 | S3 | C4 | S4 | C5 | S5 | Z score | P value |
| --- | --- | --- | --- | --- | --- | --- | --- | --- | --- | --- | --- | --- |
| Age (months) | 22 | 21 | 31 | 35 | 35 | 40 | 43 | 45 | 45 | 52 | -1.7529 | 0.0796 |
| Weight (kg) | 2.1 | 1.4 | 2.5 | 2.6 | 3.2 | 4 | 4.2 | 4.4 | 3.6 | 3.4 | -0.2709 | 0.7865 |

C: CON group; S: CUMS group. Pairs: C1/S1, C2/S2, C3/S3, C4/S4, and C5/S5.
