# Supplementary material for: Chronic unpredictable mild stress produces depressive-like behavior, hypercortisolemia, and metabolic dysfunction in adolescent cynomolgus monkeys
Source: Transl Psychiatry. 2021 Jan 4;11:9. doi: 10.1038/s41398-020-01132-6 (PMC7791128; doi:10.1038/s41398-020-01132-6)
Supplement: Supplementary file 3 — Table S2 [file 41398_2020_1132_MOESM3_ESM.docx]

**Table S2.** The definitions of chronic unpredictable mild stressors

| **Stressor** | **Definition** |
| --- | --- |
| Noise | A buzzer with a 100db shrill chirp was placed in the CUMS group rearing room for 12 hours from 8:00 PM to the next day 8:00 AM. |
| Water deprivation | The CUMS group was deprived of water supplement for 12 hours from 8:00 PM to the next day 8:00 AM. |
| Fasting | The CUMS group was deprived of food supplement for 24 hours from 8:00 AM to the next day 8:00 AM. |
| Space restriction | The cage space of CUMS group was restricted by a push-pull device for 4 hours from 8:00 AM to 12:00 AM. |
| Cold stress | The CUMS group was washed by 10℃ water for 10 min. |
| Exposure to stroboscope | The flashing stroboscopes were placed to face the CUMS group cages for 12 hours from 8:00 PM to the next day 8:00 AM. |
| Inescapable footshocks | The CUMS group was exposed to inescapable footshocks by electric shock stick (6V, 10-15s/round, 3-4 rounds, interval 10s). |

To minimize the stress duration, all mild stress procedures were completed between 8:00-12:00 AM. Apart of the stress of water deprivation and fasting, we supplied fresh fruits and vegetables every day. The researchers were not allowed to enter the room in the rest of the day. After the end of each stress, the baffles between each subject were removed to satisfy the social contacting needs of non-human primates for eight hours from 12:00 AM to 8:00 PM. Meanwhile, toys were provided for playing with each other, such as building blocks, dolls and rubber balls.
