# Supplementary material for: Chronic unpredictable mild stress produces depressive-like behavior, hypercortisolemia, and metabolic dysfunction in adolescent cynomolgus monkeys
Source: Transl Psychiatry. 2021 Jan 4;11:9. doi: 10.1038/s41398-020-01132-6 (PMC7791128; doi:10.1038/s41398-020-01132-6)
Supplement: Supplementary file 4 — Table S3 [file 41398_2020_1132_MOESM4_ESM.docx]

**Table S3.** The definitions and pictures of behavioral observations

| **Behavior** | **Definition** | **Picture** |
| --- | --- | --- |
| Huddle posture | Self- clasping with head at or below the shoulders during the waking state | ­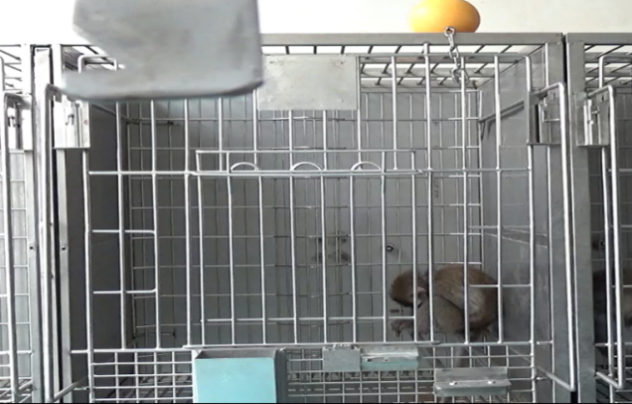 |
| Locomotion | Walking in the cage | 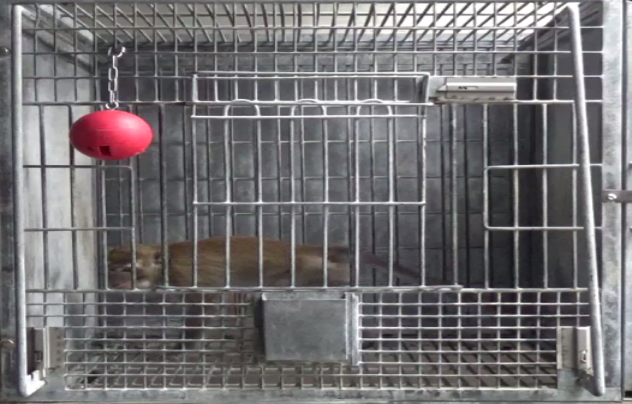 |
| Stereotypical behaviors | A repetitive motor behavior that occurs at least three times in quick succession | 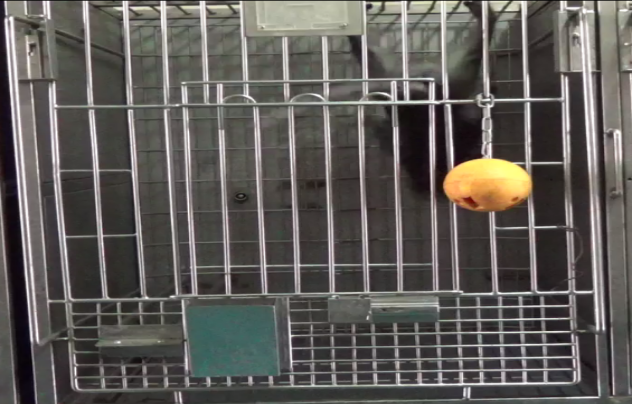 |
| Response to environment | Responding to environmental stimuli, such as feeding, drinking or exploration etc | 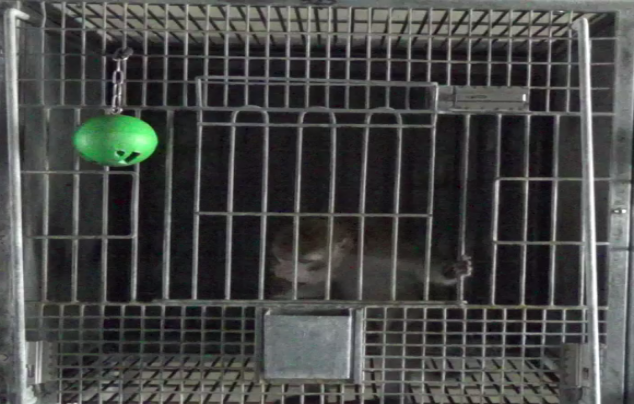 |
| Climbing | Climbing on the cage | 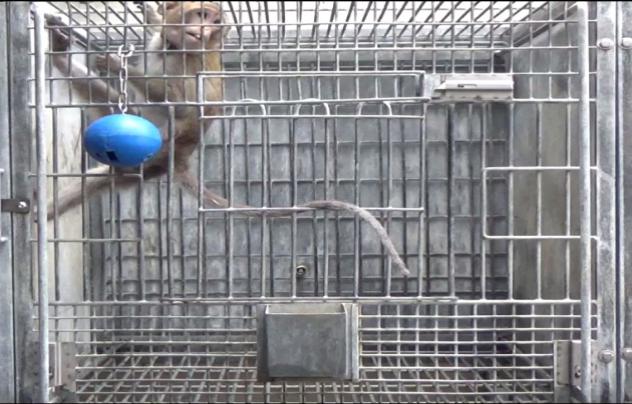 |
| Self grooming | Grooming itself | 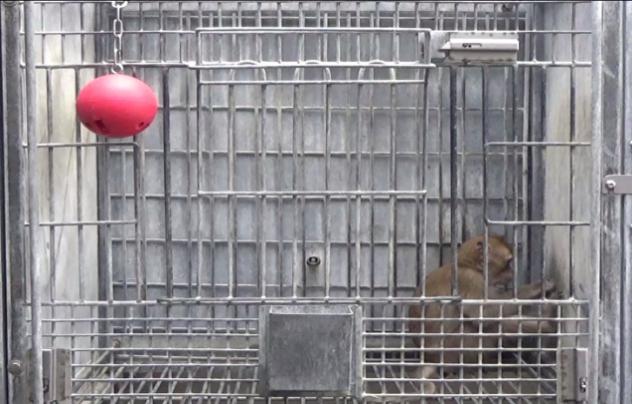 |
| Play with the ball | Playing with the ball hanging on the cage | 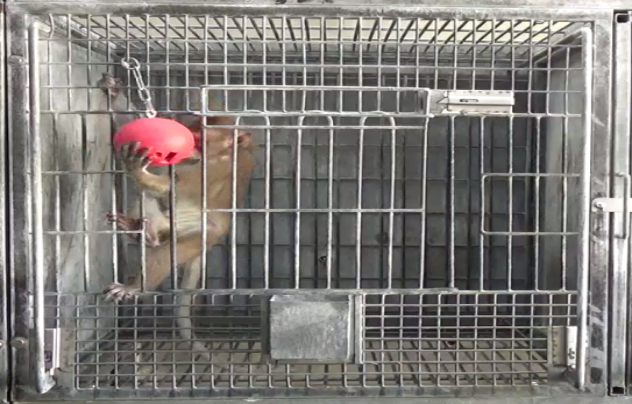 |
| Observation | Looking or trying to look the outside of cage | 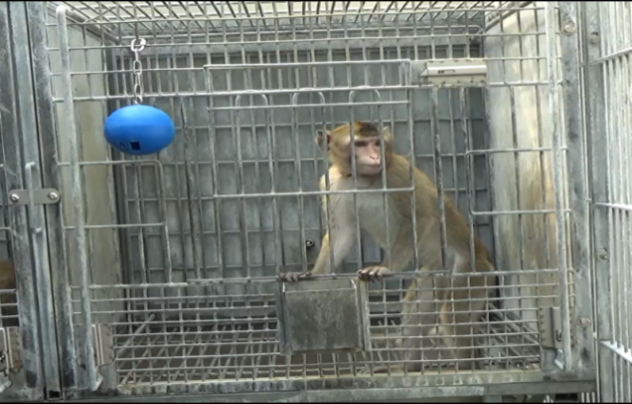 |
| Sitting | Sitting or laying on the ground | 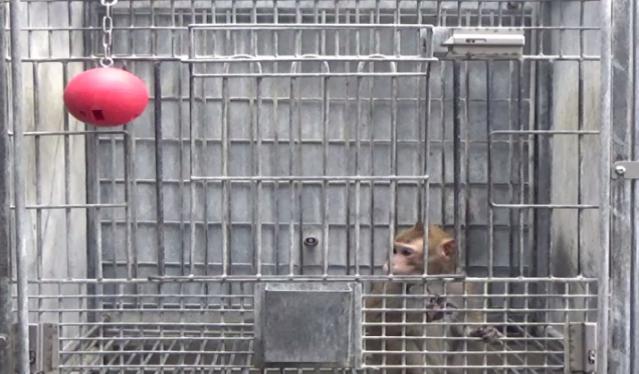 |
