# Supplementary material for: Chronic unpredictable mild stress produces depressive-like behavior, hypercortisolemia, and metabolic dysfunction in adolescent cynomolgus monkeys
Source: Transl Psychiatry. 2021 Jan 4;11:9. doi: 10.1038/s41398-020-01132-6 (PMC7791128; doi:10.1038/s41398-020-01132-6)
Supplement: Supplementary file 5 — Table S4 [file 41398_2020_1132_MOESM5_ESM.docx]

**Table S4.** The detailed procedures of attempt for apple test, human intruder test, sucrose preference test

| **Behavior test** | **Detailed procedures** |
| --- | --- |
| Attempt for apple test (AAT) | Apples were given to monkeys in the cages for 3 days adequately. Then, apples were hung out of the cages for 15 minutes during 2:00-3:00 PM for 3 days. The distance between the apple and cage was out of touching by monkeys’ arm (approximately 0.5 meters). Frequency and duration of attempting for apple were counted within 15 minutes for each day. |
| Human intruder test (HIT) | HIT consisted of four phases: (1) baseline phase: a 10-minute camera-only phase; (2) profile phase: the intruder entered the room and stood 0.6 meters from the subject’s cage for 2 minutes; (3) stare phase: the intruder turned to directly face and stare at the subject for 2 minutes without leaving the room; (4) back phase: the intruder turned directly opposite to the subject for 2 minutes without leaving the room. After the back phase, the intruder left the room and the test was complete. The last 2 minutes of baseline phase and another three phases were scored for behavioral analysis. The frequency and duration of 10 behaviors were recorded: back to the cage, pace, freeze, fear grimace, scratch, yawn, shake the cage, shake the body, self-groom and lipsmack. An anxiety category included the behaviors freeze, scratch, yawn, and fear grimace. |
| Sucrose preference test (SPT) | A four-day adaption period for 4-hour per day was conducted before the first test at baseline. At the first day, monkeys were deprived from free water supplement and obliged to drink from two pure water bottles with the same size and color. At the second day, the two pure water bottles were changed to 5% sucrose solution. At the third day, pure water placed in the right side and sucrose water in the left. At the fourth day, pure water placed in the left side and sucrose water in the right. Then, monkeys were exposed to two pre-weighted bottles with pure water or 5% sucrose water for one hour after 12 h water-deprival from 8:00 PM to the next day 8:00 AM. The preference for sucrose (%) = (sucrose amount/total amount) × 100%. |
