# Supplementary material for: Chronic unpredictable mild stress produces depressive-like behavior, hypercortisolemia, and metabolic dysfunction in adolescent cynomolgus monkeys
Source: Transl Psychiatry. 2021 Jan 4;11:9. doi: 10.1038/s41398-020-01132-6 (PMC7791128; doi:10.1038/s41398-020-01132-6)
Supplement: Supplementary file 6 — Table S5 [file 41398_2020_1132_MOESM6_ESM.docx]

**Table S5.** The definitions and pictures of behavioral tests

| **Attempt for apple test** | | |
| --- | --- | --- |
| **Behavior** | **Definition** | **Picture** |
| Attempt for apple | Trying to touch the apple | 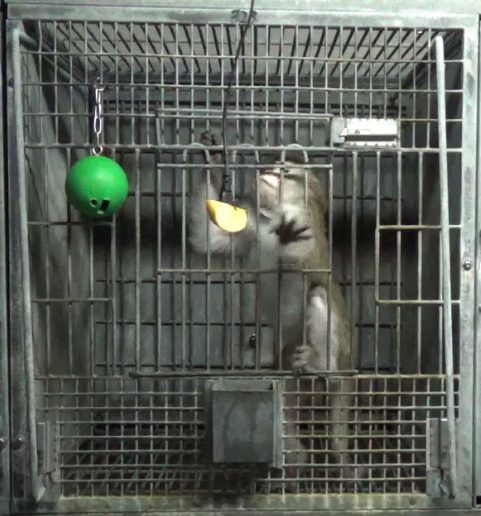 |
| **Human intruder test** | | |
| **Behavior** | **Definition** | **Picture** |
| Back to the cage | Positioning itself with at least 2 limbs in the back of the cage | 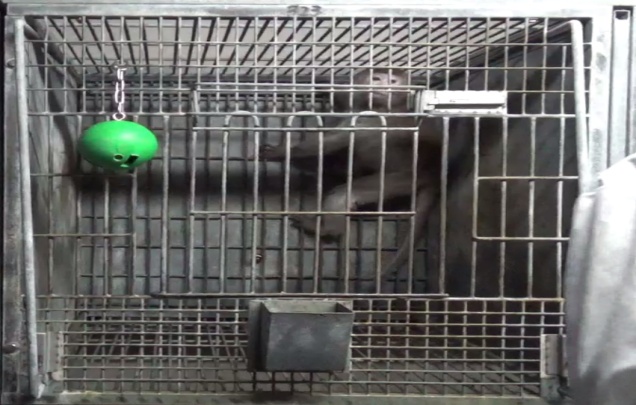 |
| Pace | Walking that exceeds 3 identical patterns | 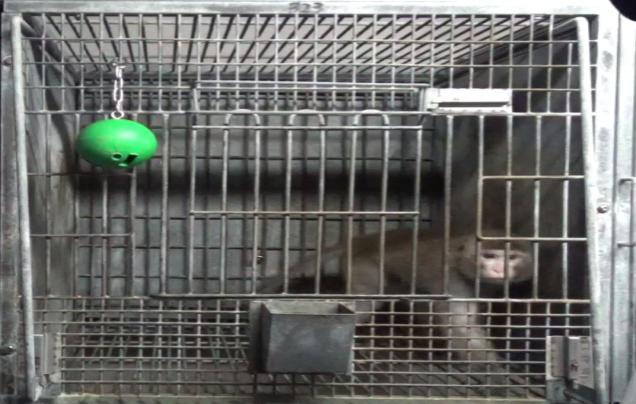 |
| Freeze | Remaining immobile for longer than 2 seconds | 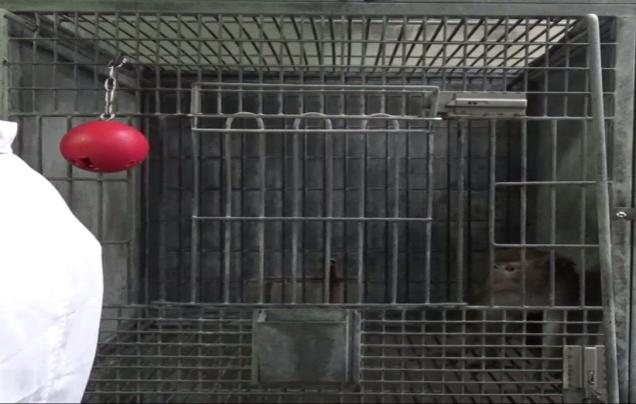 |
| Fear grimace | A large grin-like facial expression showing the teeth | 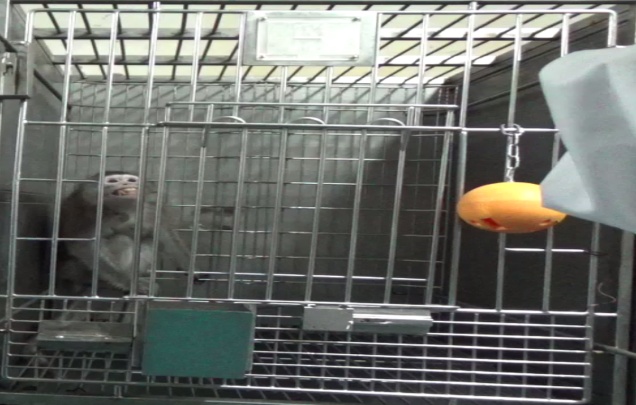 |
| Yawn | Yawn | 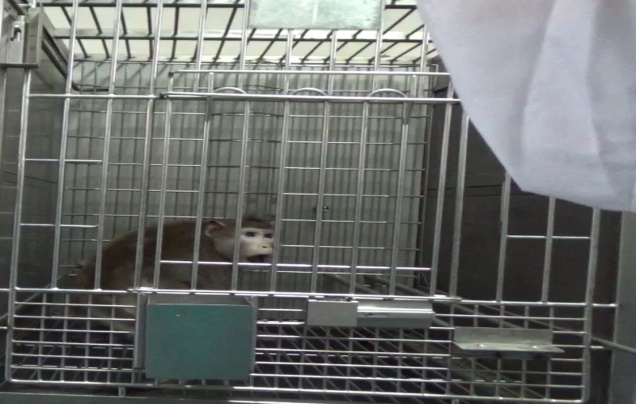 |
| Shake the cage | Grapping and shaking the cage | 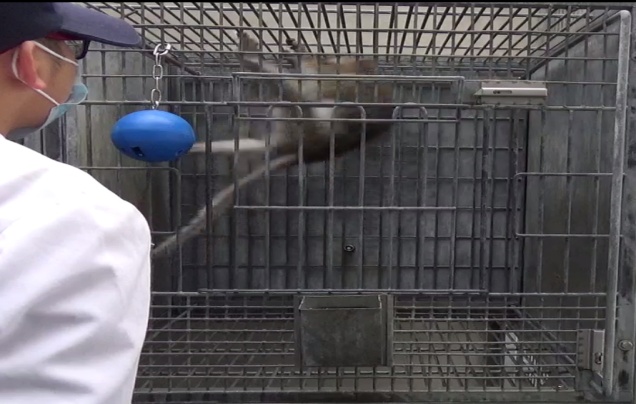 |
| Shake the body | Moving the body from one side to the other quickly | 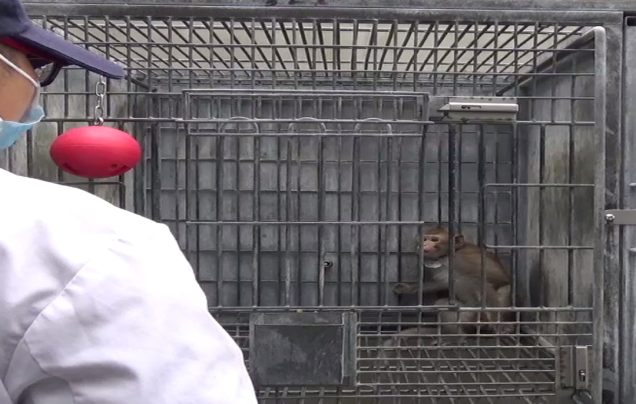 |
| Self groom | Grooming itself | 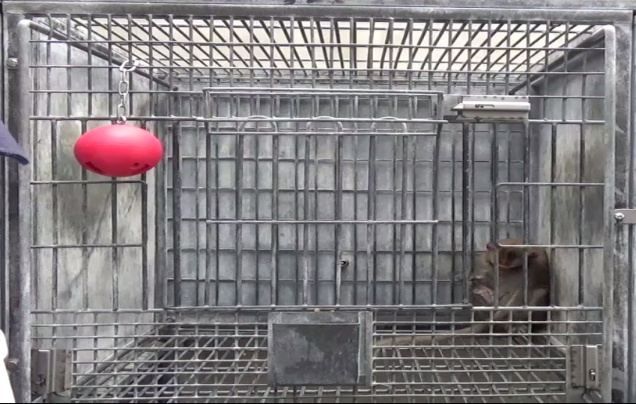 |
| Lipsmack | Opening and closing the lips rapidly | 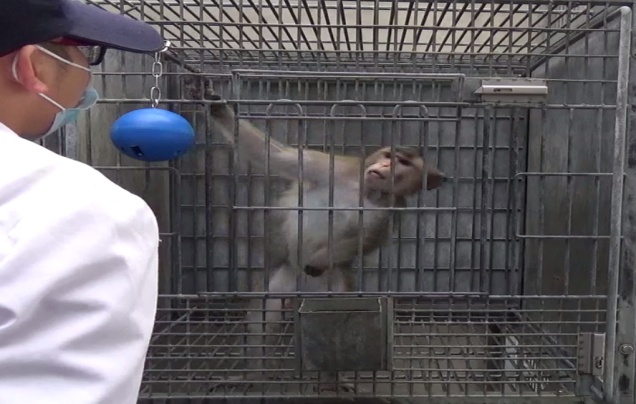 |
| Scratch | Moving repeatedly across the individual’s fur by fingertips | ‘Scratch’ was not observed in HIT. |
| **Sucrose preference test** | | |
| **Behavior** | **Definition** | **Picture** |
| Sucrose preference test | The preference for sucrose (%) = (sucrose amount/total amount) × 100% | 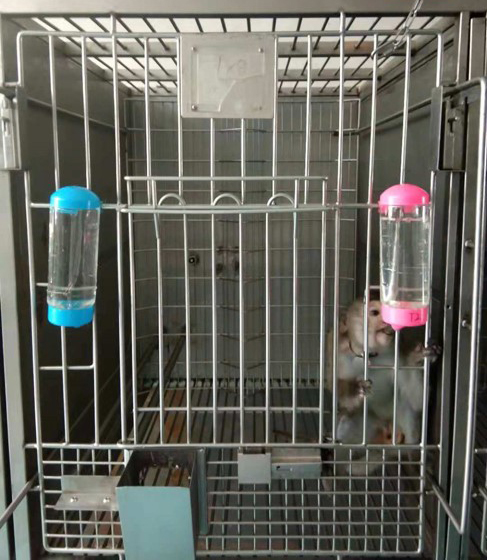 |
