# Supplementary material for: Chronic unpredictable mild stress produces depressive-like behavior, hypercortisolemia, and metabolic dysfunction in adolescent cynomolgus monkeys
Source: Transl Psychiatry. 2021 Jan 4;11:9. doi: 10.1038/s41398-020-01132-6 (PMC7791128; doi:10.1038/s41398-020-01132-6)
Supplement: Supplementary file 7 — Table S6 [file 41398_2020_1132_MOESM7_ESM.docx]

**Table S6.** The detailed results of the all the observed behaviors in CUMS (S) and CON (C) groups at baseline, midpoint (after three stress cycles) and endpoint (after five stress cycles)

|  | C1 | S1 | C2 | S2 | C3 | S3 | C4 | S4 | C5 | S5 | Z score | P value |
| --- | --- | --- | --- | --- | --- | --- | --- | --- | --- | --- | --- | --- |
| Body weight (kg) | | | | | | | | | | | | |
| Baseline | 2.1 | 1.4 | 2.5 | 2.6 | 3.2 | 4 | 4.2 | 4.4 | 3.6 | 3.4 | 0.7865 | -0.2709 |
| Midpoint | 2.1 | 1.5 | 2.7 | 2.6 | 3.2 | 4 | 4.1 | 4.4 | 3.8 | 3.5 | 1.0000 | 0.0000 |
| Endpoint | 2.1 | 1.2 | 2.6 | 2.1 | 3.3 | 3.1 | 3.9 | 3.6 | 3.6 | 3.6 | 0.0679 | -1.8257 |
| Huddle posture (frequency: times) | | | | | | | | | | | | |
| Baseline | 1 | 0 | 1 | 2 | 0 | 0 | 0 | 3 | 0 | 1 | 0.2568 | -1.1339 |
| Midpoint | 0 | 8 | 0 | 0 | 0 | 0 | 0 | 2 | 0 | 16 | 0.1088 | -1.6035 |
| Endpoint | 0 | 6 | 0 | 28 | 0 | 2 | 0 | 13 | 0 | 22 | **0.0431** | -2.0226 |
| Huddle posture (duration: seconds) | | | | | | | | | | | | |
| Baseline | 5.4 | 0.0 | 6.7 | 7.4 | 0.0 | 0.0 | 0.0 | 29.9 | 0.0 | 17.0 | 0.2733 | -1.0954 |
| Midpoint | 0.0 | 39.4 | 0.0 | 0.0 | 0.0 | 0.0 | 0.0 | 6.8 | 0.0 | 211.9 | 0.1088 | -1.6036 |
| Endpoint | 0.0 | 14.2 | 0.0 | 1070.1 | 0.0 | 16.9 | 0.0 | 656.5 | 0.0 | 345.6 | **0.0431** | -2.0226 |
| Locomotion (frequency: times) | | | | | | | | | | | | |
| Baseline | 25 | 79 | 38 | 25 | 59 | 144 | 135 | 74 | 156 | 80 | 0.8927 | -0.1348 |
| Midpoint | 172 | 92 | 48 | 97 | 81 | 89 | 149 | 74 | 142 | 83 | 0.2249 | -1.2136 |
| Endpoint | 129 | 47 | 44 | 8 | 65 | 35 | 144 | 76 | 139 | 115 | **0.0431** | -2.0226 |
| Locomotion (duration: seconds) | | | | | | | | | | | | |
| Baseline | 71.8 | 637.8 | 114.0 | 140.0 | 231.6 | 547.4 | 2291.3 | 593.9 | 398.7 | 381.9 | 0.6858 | -0.4045 |
| Midpoint | 2104.9 | 433.8 | 120.8 | 2602.8 | 756.5 | 224.2 | 1674.4 | 354.8 | 635.9 | 307.0 | 0.5002 | -0.6742 |
| Endpoint | 1018.6 | 311.3 | 83.4 | 25.6 | 235.1 | 60.2 | 1365.5 | 563.3 | 673.9 | 445.1 | **0.0431** | -2.0226 |
| Sucrose preference test (%) | | | | | | | | | | | | |
| Baseline | 60.9% | 88.6% | 82.8% | 80.4% | 86.2% | 78.8% | 81.9% | 94.2% | 58.5% | 82.0% | 0.2249 | -1.2136 |
| Midpoint | 98.8% | 96.3% | 98.6% | 99.5% | 95.7% | 98.6% | 96.2% | 99.4% | 99.1% | 98.3% | 0.3452 | -0.9439 |
| Endpoint | 93.1% | 97.9% | 96.3% | 98.6% | 94.3% | 99.2% | 99.1% | 99.4% | 99.1% | 98.8% | 0.1380 | -1.4832 |
| Stereotyped behaviors (frequency: times) | | | | | | | | | | | | |
| Baseline | 0 | 0 | 0 | 1 | 1 | 1 | 0 | 0 | 18 | 10 | 0.6547 | -0.4472 |
| Midpoint | 7 | 1 | 2 | 4 | 1 | 14 | 1 | 0 | 14 | 4 | 0.8927 | -0.1348 |
| Endpoint | 0 | 1 | 1 | 3 | 1 | 3 | 6 | 1 | 12 | 0 | 0.6845 | -0.4064 |
| Stereotyped behaviors (duration: seconds) | | | | | | | | | | | | |
| Baseline | 0.0 | 0.0 | 0.0 | 38.5 | 0.4 | 2.6 | 0.0 | 0.0 | 121.9 | 52.6 | 1.0000 | 0.0000 |
| Midpoint | 79.8 | 0.9 | 2.6 | 10.7 | 0.7 | 207.2 | 0.7 | 0.0 | 81.7 | 5.7 | 0.8927 | -0.1348 |
| Endpoint | 0.0 | 0.5 | 0.7 | 3.0 | 0.9 | 2.3 | 4.1 | 0.5 | 64.4 | 0.0 | 0.6858 | -0.4045 |
| Response behavior (frequency: times) | | | | | | | | | | | | |
| Baseline | 7 | 26 | 66 | 17 | 44 | 51 | 37 | 19 | 60 | 12 | -0.9439 | 0.3452 |
| Midpoint | 34 | 9 | 89 | 20 | 23 | 57 | 51 | 16 | 18 | 13 | -1.2136 | 0.2249 |
| Endpoint | 32 | 13 | 106 | 16 | 53 | 44 | 31 | 34 | 16 | 18 | -1.2136 | 0.2249 |
| Response behavior (duration: seconds) | | | | | | | | | | | | |
| Baseline | 238.2 | 1198.5 | 1903.1 | 773.1 | 908.5 | 597.4 | 178.3 | 111.0 | 401.9 | 52.7 | -0.9439 | 0.3452 |
| Midpoint | 149.4 | 91.7 | 1393.8 | 217.6 | 224.9 | 453.4 | 188.0 | 112.4 | 66.4 | 86.1 | -0.6742 | 0.5002 |
| Endpoint | 341.4 | 425.7 | 1220.5 | 1123.2 | 1019.3 | 1817.1 | 175.6 | 365.3 | 183.0 | 116.6 | -0.9439 | 0.3452 |
| Climb (frequency: times) | | | | | | | | | | | | |
| Baseline | 30 | 27 | 41 | 6 | 5 | 41 | 49 | 32 | 86 | 12 | -0.9439 | 0.3452 |
| Midpoint | 50 | 8 | 70 | 13 | 5 | 38 | 60 | 9 | 93 | 4 | -1.7529 | 0.0796 |
| Endpoint | 72 | 4 | 125 | 4 | 5 | 16 | 80 | 1 | 69 | 15 | 1.7529 | 0.0796 |
| Climb (duration: seconds) | | | | | | | | | | | | |
| Baseline | 2116.2 | 602.1 | 627.6 | 182.3 | 96.0 | 260.0 | 79.0 | 354.5 | 610.9 | 103.6 | -1.2136 | 0.2249 |
| Midpoint | 721.8 | 394.1 | 1214.5 | 136.0 | 32.1 | 389.8 | 145.2 | 103.9 | 845.2 | 23.9 | -1.2136 | 0.2249 |
| Endpoint | 1753.6 | 98.0 | 1288.6 | 117.8 | 94.9 | 324.9 | 213.2 | 5.9 | 766.8 | 104.6 | -1.4832 | 0.1380 |
| Self-groom (frequency: times) | | | | | | | | | | | | |
| Baseline | 17 | 15 | 27 | 19 | 1 | 22 | 24 | 26 | 6 | 2 | -0.2709 | 0.7865 |
| Midpoint | 5 | 12 | 7 | 8 | 23 | 24 | 5 | 39 | 14 | 1 | -0.9482 | 0.3430 |
| Endpoint | 4 | 7 | 12 | 17 | 2 | 12 | 4 | 8 | 2 | 0 | 1.7529 | 0.0796 |
| Self-groom (duration: seconds) | | | | | | | | | | | | |
| Baseline | 166.6 | 88.4 | 267.2 | 230.4 | 104.1 | 393.5 | 229.0 | 137.3 | 193.8 | 34.5 | -0.6742 | 0.5002 |
| Midpoint | 23.2 | 84.4 | 72.5 | 268.5 | 1154.6 | 1147.5 | 31.0 | 168.6 | 89.2 | 2.7 | -0.9439 | 0.3452 |
| Endpoint | 31.8 | 44.0 | 170.6 | 506.9 | 113.3 | 569.7 | 99.7 | 72.3 | 11.3 | 0.0 | -0.9439 | 0.3452 |
| Play with the ball (frequency: times) | | | | | | | | | | | | |
| Baseline | 1 | 1 | 0 | 3 | 0 | 3 | 0 | 3 | 0 | 0 | -1.7321 | 0.0833 |
| Midpoint | 0 | 0 | 9 | 1 | 1 | 0 | 0 | 0 | 0 | 3 | -0.5345 | 0.5930 |
| Endpoint | 3 | 0 | 5 | 0 | 0 | 0 | 0 | 0 | 0 | 0 | -1.3416 | 0.1797 |
| Play with the ball (duration: seconds) | | | | | | | | | | | | |
| Baseline | 6.6 | 5.2 | 0.0 | 51.1 | 0.0 | 15.6 | 0.0 | 32.9 | 0.0 | 0.0 | -1.4606 | 0.1441 |
| Midpoint | 0.0 | 0.0 | 76.1 | 6.1 | 18.4 | 0.0 | 0.0 | 0.0 | 0.0 | 18.6 | -0.5345 | 0.5930 |
| Endpoint | 10.8 | 0.0 | 97.5 | 0.0 | 0.0 | 0.0 | 0.0 | 0.0 | 0.0 | 0.0 | -1.3416 | 0.1797 |
| Observation (frequency: times) | | | | | | | | | | | | |
| Baseline | 19 | 40 | 13 | 11 | 21 | 58 | 86 | 35 | 82 | 51 | -0.4045 | 0.6858 |
| Midpoint | 114 | 68 | 13 | 68 | 68 | 48 | 101 | 65 | 99 | 58 | -0.6742 | 0.5002 |
| Endpoint | 105 | 21 | 20 | 23 | 27 | 3 | 96 | 39 | 86 | 76 | 1.7529 | 0.0796 |
| Observation (duration: seconds) | | | | | | | | | | | | |
| Baseline | 220.9 | 110.2 | 302.9 | 241.3 | 439.5 | 123.4 | 119.7 | 220.1 | 108.4 | 272.7 | -1.7529 | 0.0796 |
| Midpoint | 307.3 | 29.6 | 387.3 | 1261.9 | 530.4 | 92.9 | 214.1 | 205.9 | 468.9 | 182.4 | -1.4832 | 0.1380 |
| Endpoint | 316.8 | 89.8 | 319.0 | 682.8 | 521.2 | 166.6 | 462.3 | 8.8 | 150.5 | 292.2 | -0.9439 | 0.3452 |
| Sit down (frequency: times) | | | | | | | | | | | | |
| Baseline | 23 | 40 | 45 | 27 | 38 | 31 | 37 | 70 | 69 | 48 | -1.7529 | 0.0796 |
| Midpoint | 18 | 22 | 20 | 27 | 51 | 45 | 17 | 69 | 69 | 64 | -1.7529 | 0.0796 |
| Endpoint | 15 | 25 | 53 | 37 | 22 | 42 | 37 | 27 | 38 | 76 | -1.2136 | 0.2249 |
| Sit down (duration: seconds) | | | | | | | | | | | | |
| Baseline | 774.2 | 944.6 | 571.1 | 2085.8 | 1956.7 | 1564.1 | 581.4 | 2232.2 | 1433.6 | 2674.1 | -1.4832 | 0.1380 |
| Midpoint | 213.7 | 2462.9 | 219.1 | 144.4 | 436.3 | 972.2 | 299.1 | 2385.0 | 1351.4 | 2762.4 | 1.7529 | 0.0796 |
| Endpoint | 127.3 | 2539.7 | 648.6 | 291.3 | 1817.5 | 692.0 | 1018.4 | 1785.6 | 1379.9 | 2174.7 | -0.6742 | 0.5002 |

C: CON group; S: CUMS group. Pairs: C1/S1, C2/S2, C3/S3, C4/S4, and C5/S5. The frequency means the number of times of the behaviors happening in the one-hour videotape. The duration means the total seconds of the behaviors happening in the one-hour videotape.
