# Supplementary material for: Chronic unpredictable mild stress produces depressive-like behavior, hypercortisolemia, and metabolic dysfunction in adolescent cynomolgus monkeys
Source: Transl Psychiatry. 2021 Jan 4;11:9. doi: 10.1038/s41398-020-01132-6 (PMC7791128; doi:10.1038/s41398-020-01132-6)
Supplement: Supplementary file 8 — Table S7 [file 41398_2020_1132_MOESM8_ESM.docx]

**Table S7.** The detailed results of the attempt for apple test, and the behaviors in each phase of human intruder test in CUMS (S) and CON (S) groups

| **Attempt for apple test** | | | | | | | | | | | | |
| --- | --- | --- | --- | --- | --- | --- | --- | --- | --- | --- | --- | --- |
|  | C1 | S1 | C2 | S2* | C3 | S3 | C4 | S4 | C5 | S5 | Z score | P value |
| Attempt for apple (frequency: times) | | | | | | | | | | | | |
| Day 1 | 0 | 4 | 3 | 6.5 | 27 | 11 | 4 | 7 | 4 | 6 | -0.6742 | 0.5002 |
| Day 2 | 2 | 0 | 4 | 0 | 1 | 0 | 0 | 0 | 0 | 0 | -1.6036 | 0.1088 |
| Day 3 | 8 | 0 | 0 | 0 | 6 | 0 | 0 | 0 | 0 | 0 | -1.3416 | 0.1797 |
| Total | 10 | 4 | 7 | 6.5 | 34 | 11 | 4 | 7 | 4 | 6 | -0.6742 | 0.5002 |
| Attempt for apple (duration: seconds) | | | | | | | | | | | | |
| Day 1 | 0.0 | 10.9 | 6.2 | 11.1 | 89.5 | 21.9 | 13.0 | 11.4 | 11.3 | 8.7 | -0.1348 | 0.8927 |
| Day 2 | 10.4 | 0.0 | 6.4 | 0.0 | 1.7 | 0.0 | 0.0 | 0.0 | 0.0 | 0.0 | -1.6036 | 0.1088 |
| Day 3 | 7.4 | 0.0 | 0.0 | 0.0 | 18.9 | 0.0 | 0.0 | 0.0 | 0.0 | 0.0 | -1.3416 | 0.1797 |
| Total | 17.8 | 10.9 | 12.6 | 11.1 | 110.1 | 21.9 | 13.0 | 11.4 | 11.3 | 8.7 | -2.0226 | **0.0431** |
| **Human intruder test** | | | | | | | | | | | | |
|  | C1 | S1 | C2 | S2* | C3 | S3 | C4 | S4 | C5 | S5 | Z score | P value |
| Freeze (frequency: times) | | | | | | | | | | | | |
| Baseline | 2 | 0 | 3 | 0 | 3 | 5 | 1 | 0 | 5 | 0 | -1.3546 | 0.1756 |
| Profile | 0 | 2 | 3 | 1 | 4 | 9 | 0 | 0 | 2 | 0 | -0.3780 | 0.7055 |
| Stare | 0 | 0 | 0 | 0 | 0 | 0 | 0 | 0 | 1 | 0 | -1.0000 | 0.3173 |
| Back | 0 | 0 | 1 | 0 | 0 | 2 | 0 | 0 | 0 | 0 | -0.4472 | 0.6547 |
| Total | 2 | 2 | 7 | 1 | 7 | 16 | 1 | 0 | 8 | 0 | -0.3651 | 0.7150 |
| Freeze (duration: seconds) | | | | | | | | | | | | |
| Baseline | 10.9 | 0.0 | 10.3 | 0.0 | 10.2 | 20.5 | 2.5 | 0.0 | 21.2 | 0.0 | -1.4832 | 0.1380 |
| Profile | 0.0 | 4.2 | 11.0 | 2.1 | 24.6 | 35.8 | 0.0 | 0.0 | 29.4 | 0.0 | -0.3651 | 0.7150 |
| Stare | 0.0 | 0.0 | 0.0 | 0.0 | 0.0 | 0.0 | 0.0 | 0.0 | 5.4 | 0.0 | -1.0000 | 0.3173 |
| Back | 0.0 | 0.0 | 2.2 | 0.0 | 0.0 | 6.5 | 0.0 | 0.0 | 0.0 | 0.0 | -0.4472 | 0.6547 |
| Total | 10.9 | 4.2 | 23.5 | 2.1 | 34.8 | 62.7 | 2.5 | 0.0 | 56.0 | 0.0 | -0.9439 | 0.3452 |
| Yawn (frequency: times) | | | | | | | | | | | | |
| Baseline | 0 | 0 | 0 | 0 | 1 | 0 | 0 | 0 | 0 | 0 | -1.0000 | 0.3173 |
| Profile | 0 | 0 | 0 | 0 | 0 | 0 | 2 | 1 | 2 | 0 | -1.3416 | 0.1797 |
| Stare | 0 | 1 | 0 | 0.5 | 0 | 10 | 3 | 0 | 1 | 0 | -0.2709 | 0.7865 |
| Back | 0 | 0 | 0 | 0 | 5 | 0 | 4 | 0 | 0 | 0 | -1.3416 | 0.1797 |
| Total | 0 | 1 | 0 | 0.5 | 6 | 10 | 9 | 1 | 3 | 0 | -0.1348 | 0.8927 |
| Yawn (duration: seconds) | | | | | | | | | | | | |
| Baseline | 0.0 | 0.0 | 0.0 | 0.0 | 1.7 | 0.0 | 0.0 | 0.0 | 0.0 | 0.0 | -1.0000 | 0.3173 |
| Profile | 0.0 | 0.0 | 0.0 | 0.0 | 0.0 | 0.0 | 1.5 | 1.8 | 0.9 | 0.0 | -0.4472 | 0.6547 |
| Stare | 0.0 | 0.1 | 0.0 | 0.1 | 0.0 | 6.8 | 1.9 | 0.0 | 1.4 | 0.0 | -0.1348 | 0.8927 |
| Back | 0.0 | 0.0 | 0.0 | 0.0 | 6.2 | 0.0 | 6.5 | 0.0 | 0.0 | 0.0 | -1.3416 | 0.1797 |
| Total | 0.0 | 0.1 | 0.0 | 0.1 | 7.9 | 6.8 | 9.9 | 1.8 | 2.3 | 0.0 | -1.2136 | 0.2249 |
| Fear grimace (frequency: times) | | | | | | | | | | | | |
| Baseline | 0 | 0 | 0 | 0 | 0 | 0 | 0 | 0 | 0 | 0 | 0.0000 | 1.0000 |
| Profile | 0 | 0 | 0 | 0 | 0 | 0 | 0 | 5 | 0 | 0 | -1.0000 | 0.3173 |
| Stare | 0 | 0 | 0 | 4.5 | 0 | 7 | 0 | 24 | 4 | 2 | -1.4606 | 0.1441 |
| Back | 0 | 0 | 0 | 0 | 1 | 0 | 4 | 10 | 0 | 0 | -0.4472 | 0.6547 |
| Total | 0 | 0 | 0 | 4.5 | 1 | 7 | 4 | 39 | 4 | 2 | -1.4606 | 0.1441 |
| Fear grimace (duration: seconds) | | | | | | | | | | | | |
| Baseline | 0.0 | 0.0 | 0.0 | 0.0 | 0.0 | 0.0 | 0.0 | 0.0 | 0.0 | 0.0 | 0.0000 | 1.0000 |
| Profile | 0.0 | 0.0 | 0.0 | 0.0 | 0.0 | 0.0 | 0.0 | 47.6 | 0.0 | 0.0 | -1.0000 | 0.3173 |
| Stare | 0.0 | 0.0 | 0.0 | 16.9 | 0.0 | 9.2 | 0.0 | 24.5 | 7.9 | 35.3 | -1.8257 | 0.0679 |
| Back | 0.0 | 0.0 | 0.0 | 0.0 | 1.9 | 0.0 | 7.1 | 4.6 | 0.0 | 0.0 | -1.3416 | 0.1797 |
| Total | 0.0 | 0.0 | 0.0 | 16.9 | 1.9 | 9.2 | 7.1 | 76.7 | 7.9 | 35.3 | -1.8257 | 0.0679 |
| Back of cage (frequency: times) | | | | | | | | | | | | |
| Baseline | 1 | 0 | 0 | 0 | 5 | 0 | 1 | 0 | 0 | 0 | -1.6330 | 0.1025 |
| Profile | 2 | 4 | 2 | 1 | 1 | 0 | 2 | 1 | 0 | 1 | 0.0000 | 1.0000 |
| Stare | 4 | 10 | 2 | 1 | 1 | 0 | 1 | 1 | 0 | 1 | -0.3780 | 0.7055 |
| Back | 0 | 0 | 2 | 0 | 1 | 0 | 3 | 0 | 0 | 4 | -0.3651 | 0.7150 |
| Total | 7 | 14 | 6 | 2 | 8 | 0 | 7 | 2 | 0 | 6 | -0.1348 | 0.8927 |
| Back of cage (duration: seconds) | | | | | | | | | | | | |
| Baseline | 10.9 | 0.0 | 0.0 | 0.0 | 88.0 | 0.0 | 5.2 | 0.0 | 0.0 | 0.0 | -1.6036 | 0.1088 |
| Profile | 18.2 | 35.3 | 114.4 | 76.2 | 120.0 | 0.0 | 116.3 | 117.0 | 0.0 | 120.5 | -0.1348 | 0.8927 |
| Stare | 14.4 | 20.9 | 118.0 | 70.5 | 119.6 | 0.0 | 120.1 | 120.1 | 0.0 | 120.4 | -0.1348 | 0.8927 |
| Back | 0.0 | 0.0 | 109.3 | 0.0 | 120.1 | 0.0 | 112.5 | 0.0 | 0.0 | 92.3 | -1.4606 | 0.1441 |
| Total | 43.5 | 56.3 | 341.6 | 146.7 | 447.8 | 0.0 | 354.0 | 237.1 | 0.0 | 333.1 | -0.6742 | 0.5002 |
| Shake the cage (frequency: times) | | | | | | | | | | | | |
| Baseline | 0 | 0 | 0 | 0 | 0 | 0 | 0 | 0 | 0 | 0 | 0.0000 | 1.0000 |
| Profile | 0 | 0 | 0 | 0 | 0 | 0 | 0 | 0 | 0 | 0 | 0.0000 | 1.0000 |
| Stare | 0 | 0 | 0 | 0 | 0 | 0 | 0 | 0 | 2 | 0 | -1.0000 | 0.3173 |
| Back | 0 | 0 | 0 | 0 | 0 | 0 | 0 | 0 | 0 | 0 | 0.0000 | 1.0000 |
| Total | 0 | 0 | 0 | 0 | 0 | 0 | 0 | 0 | 2 | 0 | -1.0000 | 0.3173 |
| Shake the cage (duration: seconds) | | | | | | | | | | | | |
| Baseline | 0.0 | 0.0 | 0.0 | 0.0 | 0.0 | 0.0 | 0.0 | 0.0 | 0.0 | 0.0 | 0.0000 | 1.0000 |
| Profile | 0.0 | 0.0 | 0.0 | 0.0 | 0.0 | 0.0 | 0.0 | 0.0 | 0.0 | 0.0 | 0.0000 | 1.0000 |
| Stare | 0.0 | 0.0 | 0.0 | 0.0 | 0.0 | 0.0 | 0.0 | 0.0 | 5.4 | 0.0 | -1.0000 | 0.3173 |
| Back | 0.0 | 0.0 | 0.0 | 0.0 | 0.0 | 0.0 | 0.0 | 0.0 | 0.0 | 0.0 | 0.0000 | 1.0000 |
| Total | 0.0 | 0.0 | 0.0 | 0.0 | 0.0 | 0.0 | 0.0 | 0.0 | 5.4 | 0.0 | -1.0000 | 0.3173 |
| Self-groom (frequency: times) | | | | | | | | | | | | |
| Baseline | 0 | 0 | 0 | 0 | 0 | 0 | 0 | 0 | 1 | 0 | -1.0000 | 0.3173 |
| Profile | 0 | 0 | 0 | 0 | 0 | 0 | 0 | 0 | 0 | 0 | 0.0000 | 1.0000 |
| Stare | 0 | 0 | 0 | 0 | 0 | 0 | 0 | 0 | 0 | 0 | 0.0000 | 1.0000 |
| Back | 0 | 0 | 0 | 0 | 0 | 0 | 1 | 0 | 0 | 0 | -1.0000 | 0.3173 |
| Total | 0 | 0 | 0 | 0 | 0 | 0 | 1 | 0 | 1 | 0 | -1.4142 | 0.1573 |
| Self-groom (duration: seconds) | | | | | | | | | | | | |
| Baseline | 0.0 | 0.0 | 0.0 | 0.0 | 0.0 | 0.0 | 0.0 | 0.0 | 4.1 | 0.0 | -1.0000 | 0.3173 |
| Profile | 0.0 | 0.0 | 0.0 | 0.0 | 0.0 | 0.0 | 0.0 | 0.0 | 0.0 | 0.0 | 0.0000 | 1.0000 |
| Stare | 0.0 | 0.0 | 0.0 | 0.0 | 0.0 | 0.0 | 0.0 | 0.0 | 0.0 | 0.0 | 0.0000 | 1.0000 |
| Back | 0.0 | 0.0 | 0.0 | 0.0 | 0.0 | 0.0 | 9.9 | 0.0 | 0.0 | 0.0 | -1.0000 | 0.3173 |
| Total | 0.0 | 0.0 | 0.0 | 0.0 | 0.0 | 0.0 | 9.9 | 0.0 | 4.1 | 0.0 | -1.3416 | 0.1797 |
| Lipsmack (frequency: times) | | | | | | | | | | | | |
| Baseline | 0 | 0 | 0 | 0 | 0 | 0 | 0 | 3 | 0 | 0 | -1.0000 | 0.3173 |
| Profile | 2 | 1 | 3 | 1 | 0 | 0 | 1 | 2 | 0 | 1 | -0.3780 | 0.7055 |
| Stare | 8 | 12 | 1 | 2 | 2 | 3 | 1 | 1 | 5 | 1 | -0.5571 | 0.5775 |
| Back | 0 | 0 | 1 | 0 | 0 | 0 | 1 | 0 | 0 | 0 | -1.4142 | 0.1573 |
| Total | 10 | 13 | 5 | 3 | 2 | 3 | 3 | 6 | 5 | 2 | -0.4121 | 0.6803 |
| Lipsmack (duration: seconds) | | | | | | | | | | | | |
| Baseline | 0.0 | 0.0 | 0.0 | 0.0 | 0.0 | 0.0 | 0.0 | 11.4 | 0.0 | 0.0 | -1.0000 | 0.3173 |
| Profile | 1.0 | 1.3 | 13.6 | 10.3 | 0.0 | 0.0 | 2.2 | 116.9 | 0.0 | 19.2 | -1.0954 | 0.2733 |
| Stare | 76.1 | 68.3 | 107.6 | 94.2 | 94.8 | 30.6 | 119.9 | 120.1 | 42.3 | 120.4 | -0.4045 | 0.6858 |
| Back | 0.0 | 0.0 | 4.2 | 0.0 | 0.0 | 0.0 | 2.9 | 0.0 | 0.0 | 0.0 | -1.3416 | 0.1797 |
| Total | 77.1 | 69.6 | 125.5 | 104.5 | 94.8 | 30.6 | 125.1 | 248.3 | 42.3 | 139.6 | -0.4045 | 0.6858 |
| Shake the cage (frequency: times) | | | | | | | | | | | | |
| Baseline | 0 | 0 | 0 | 0 | 0 | 0 | 0 | 0 | 0 | 0 | 0.0000 | 1.0000 |
| Profile | 0 | 0 | 0 | 0 | 0 | 0 | 1 | 0 | 0 | 0 | -1.0000 | 0.3173 |
| Stare | 0 | 0 | 0 | 0 | 0 | 0 | 4 | 0 | 1 | 0 | -1.3416 | 0.1797 |
| Back | 0 | 0 | 0 | 0 | 0 | 0 | 1 | 0 | 0 | 0 | -1.0000 | 0.3173 |
| Total | 0 | 0 | 0 | 0 | 0 | 0 | 6 | 0 | 1 | 0 | -1.3416 | 0.1797 |
| Shake the cage (duration: seconds) | | | | | | | | | | | | |
| Baseline | 0.0 | 0.0 | 0.0 | 0.0 | 0.0 | 0.0 | 0.0 | 0.0 | 0.0 | 0.0 | 0.0000 | 1.0000 |
| Profile | 0.0 | 0.0 | 0.0 | 0.0 | 0.0 | 0.0 | 2.7 | 0.0 | 0.0 | 0.0 | -1.0000 | 0.3173 |
| Stare | 0.0 | 0.0 | 0.0 | 0.0 | 0.0 | 0.0 | 68.4 | 0.0 | 6.4 | 0.0 | -1.3416 | 0.1797 |
| Back | 0.0 | 0.0 | 0.0 | 0.0 | 0.0 | 0.0 | 8.2 | 0.0 | 0.0 | 0.0 | -1.0000 | 0.3173 |
| Total | 0.0 | 0.0 | 0.0 | 0.0 | 0.0 | 0.0 | 79.3 | 0.0 | 6.4 | 0.0 | -1.3416 | 0.1797 |
| Pace (frequency: times) | | | | | | | | | | | | |
| Baseline | 8 | 21 | 1 | 7 | 1 | 12 | 1 | 2 | 0 | 2 | -2.0226 | **0.0431** |
| Profile | 11 | 5 | 0 | 0 | 0 | 0 | 0 | 0 | 1 | 0 | -1.3416 | 0.1797 |
| Stare | 13 | 16 | 0 | 0 | 0 | 0 | 1 | 0 | 0 | 0 | -0.4472 | 0.6547 |
| Back | 10 | 3 | 0 | 2.5 | 0 | 3 | 3 | 0 | 2 | 2 | -0.5523 | 0.5807 |
| Total | 42 | 45 | 1 | 9.5 | 1 | 15 | 5 | 2 | 3 | 4 | -1.3546 | 0.1756 |
| Pace (duration: seconds) | | | | | | | | | | | | |
| Baseline | 39.5 | 74.6 | 2.7 | 31.7 | 1.6 | 58.0 | 1.3 | 4.3 | 0.0 | 5.5 | -2.0226 | **0.0431** |
| Profile | 69.7 | 26.6 | 0.0 | 0.0 | 0.0 | 0.0 | 0.0 | 0.0 | 3.6 | 0.0 | -1.3416 | 0.1797 |
| Stare | 44.4 | 18.5 | 0.0 | 0.0 | 0.0 | 0.0 | 2.1 | 0.0 | 0.0 | 0.0 | -1.3416 | 0.1797 |
| Back | 102.4 | 115.9 | 0.0 | 5.7 | 0.0 | 6.4 | 4.5 | 0.0 | 7.5 | 5.0 | -1.2136 | 0.2249 |
| Total | 255.9 | 235.6 | 2.7 | 37.4 | 1.6 | 64.3 | 8.0 | 4.3 | 11.1 | 10.5 | -0.4045 | 0.6858 |

C: CON group; S: CUMS group. Pairs: C1/S1, C2/S2, C3/S3, C4/S4, and C5/S5. Significant results are bolded and underscored. The frequency means the number of times of the behaviors happening in the one-hour videotape. The duration means the total seconds of the behaviors happening in the one-hour videotape.

* Subject S2 in CUMS group dropped out of AAT and HIT due to diarrhea, so we imputed the median value for each behavior in CUMS group as the value of S2 in statistical analysis.
