# Supplementary material for: Chronic unpredictable mild stress produces depressive-like behavior, hypercortisolemia, and metabolic dysfunction in adolescent cynomolgus monkeys
Source: Transl Psychiatry. 2021 Jan 4;11:9. doi: 10.1038/s41398-020-01132-6 (PMC7791128; doi:10.1038/s41398-020-01132-6)
Supplement: Supplementary file 9 — Table S8 [file 41398_2020_1132_MOESM9_ESM.docx]

**Table S8.** The levels of hair and plasma cortisol in CUMS (S) group and CON (C) group at baseline and endpoint (after five stress cycles)

|  | C1 | S1 | C2 | S2 | C3 | S3 | C4 | S4 | C5 | S5 | Z score | P value |
| --- | --- | --- | --- | --- | --- | --- | --- | --- | --- | --- | --- | --- |
| **Baseline** | | | | | | | | | | | | |
| Hair cortisol (ng/ml) | 40.74 | 56.91 | 44.44 | 57.61 | 38.90 | 51.60 | 74.01 | 22.03 | 66.85 | 13.72 | -1.2136 | 0.2249 |
| Plasma cortisol (ng/ml) | 108.19 | 164.32 | 138.22 | 94.10 | 77.47 | 205.44 | 180.26 | 164.55 | 119.97 | 176.33 | -0.4045 | 0.6858 |
| **Endpoint** | | | | | | | | | | | | |
| Hair cortisol (ng/ml) | 65.8 | 92.3 | 71.6 | 80.8 | 83.0 | 91.9 | 69.1 | 83.3 | 58.7 | 92.3 | -2.0226 | **0.0431** |
| Plasma cortisol (ng/ml) | 107.9 | 151.3 | 98.6 | 176.9 | 131.2 | 119.1 | 181.0 | 99.1 | 185.5 | 166.6 | -0.1348 | 0.8927 |

C: CON group; S: CUMS group; Pairs: C1/S1, C2/S2, C3/S3, C4/S4, and C5/S5. Significant results are bolded and underscored.
