# Supplementary material for: Chronic unpredictable mild stress produces depressive-like behavior, hypercortisolemia, and metabolic dysfunction in adolescent cynomolgus monkeys
Source: Transl Psychiatry. 2021 Jan 4;11:9. doi: 10.1038/s41398-020-01132-6 (PMC7791128; doi:10.1038/s41398-020-01132-6)
Supplement: Supplementary file 10 — Table S9 [file 41398_2020_1132_MOESM10_ESM.docx]

**Table S9.** Identified differential metabolites in the plasma between the CUMS and CON groups

| **Metabolites** | **ESC^+/-^** | **HMDB** | **P-value** | **FC^a^** | **VIP** |
| --- | --- | --- | --- | --- | --- |
| 3-Methoxy-4-Hydroxyphenylglycol sulfate | - | HMDB0003332 | 0.0431 | 1.77↑ | 1.60 |
| Phosphoadenosine phosphosulfate | + | HMDB0001134 | 0.0431 | 0.35↓ | 1.83 |
| L-Acetylcarnitine | + | HMDB0000201 | 0.0431 | 0.87↓ | 1.25 |
| Acetylvalerenolic acid | - | HMDB0035687 | 0.0431 | 0.51↓ | 1.34 |
| Phosphoadenosine phosphate | + | HMDB0000061 | 0.0431 | 0.48↓ | 1.67 |
| Allocystathionine | + | HMDB0000455 | 0.0431 | 0.53↓ | 1.54 |
| Galactose 1-phosphate | - | HMDB0000645 | 0.0431 | 0.36↓ | 1.55 |
| Arginyl-Glutamate | + | HMDB0028708 | 0.0431 | 0.54↓ | 1.83 |
| Cholic acid | - | HMDB0000619 | 0.0431 | 2.55↑ | 1.50 |
| Citric acid | - | HMDB0000094 | 0.0431 | 0.51↓ | 1.41 |
| Cyclohexylamine | + | HMDB0031404 | 0.0431 | 0.43↓ | 1.77 |
| dGDP | - | HMDB0000960 | 0.0431 | 0.41↓ | 1.61 |
| dGTP | - | HMDB0001440 | 0.0431 | 0.29↓ | 1.81 |
| D-Glucaric acid | - | HMDB0029881 | 0.0431 | 0.46↓ | 1.37 |
| Glycerone | - | HMDB0001882 | 0.0431 | 0.54↓ | 1.44 |
| D-Mannose | + | HMDB0000169 | 0.0431 | 0.85↓ | 1.31 |
| D-Ornithine | - | HMDB0003374 | 0.0431 | 1.95↑ | 1.67 |
| Ethyl (+/-)-3-hydroxybutyrate | + | HMDB40409 | 0.0431 | 1.39↑ | 1.41 |
| gamma-Glutamylglutamic acid | + | HMDB0011737 | 0.0431 | 0.73↓ | 1.46 |
| Glyceric acid | - | HMDB0000139 | 0.0431 | 0.43↓ | 1.95 |
| Histidinyl-Proline | + | HMDB0028893 | 0.0431 | 0.34↓ | 1.47 |
| Hydroxyisocaproic acid | - | HMDB0000746 | 0.0431 | 0.40↓ | 1.06 |
| L-Methionine | - | HMDB0000696 | 0.0431 | 0.46↓ | 1.45 |
| Pyroglutamic acid | + | HMDB0000267 | 0.0431 | 0.11↓ | 1.28 |
| Malonic acid | - | HMDB0000691 | 0.0431 | 0.35↓ | 1.89 |
| N-Acetylornithine | + | HMDB0003357 | 0.0431 | 0.54↓ | 1.26 |
| SM(d18:1/18:0) | + | HMDB0001348 | 0.0431 | 0.27↓ | 1.31 |
| Stearic acid | - | HMDB0000827 | 0.0431 | 1.28↑ | 1.40 |
| Uridine diphosphate-N-acetylglucosamine | + | HMDB0000290 | 0.0431 | 1.36↑ | 1.45 |
| Urocanic acid | - | HMDB0000301 | 0.0431 | 1.80↑ | 1.30 |

^a^ Fold Change was calculated as the ratio of the average variable value in the CUMS group to that in the CON group.

↑: up- regulated in CUMS group; ↓: down-regulated in CUMS group.

VIP: variable importance on projection; dGDP: deoxyguanosine diphosphate; dGTP: deoxyguanosine triphosphate; SM: Sphingomyelin.
