# Supplementary material for: Chronic unpredictable mild stress produces depressive-like behavior, hypercortisolemia, and metabolic dysfunction in adolescent cynomolgus monkeys
Source: Transl Psychiatry. 2021 Jan 4;11:9. doi: 10.1038/s41398-020-01132-6 (PMC7791128; doi:10.1038/s41398-020-01132-6)
Supplement: Supplementary file 11 — Table S10 [file 41398_2020_1132_MOESM11_ESM.docx]

**Table S10.** The significantly altered pathways of depression in adolescent monkeys and patients

| Depression in adolescent monkeys | | |  | Depression in adolescent patients^#^ | | |
| --- | --- | --- | --- | --- | --- | --- |
| Pathways* | Metabolites | P-value |  | Pathways* | Metabolites | P-value |
| Sulfur metabolism | Phosphoadenosine phosphosulfate, allocystathionine, phosphoadenosine phosphate | 6.45E-04 |  | Fatty acid biosynthesis | Dodecanoic acid, palmitoleic acid, capric acid; oleic acid, palmitic acid | 5.77E-05 |
| Purine metabolism | dGDP, dGTP, phosphoadenosine phosphosulfate, phosphoadenosine phosphate | 1.18E-02 |  | Arginine and proline metabolism | L-Arginine, creatine, creatinine; | 3.13E-02 |
| Glycerolipid metabolism | Glycerone, glyceric acid | 3.94E-02 |  | Purine metabolism | Adenosine, inosine, hypoxanthine; | 4.92E-02 |

*The altered pathways were calculated by MetaboAnalyst.

#The results were from our previous study (Zhou X, Liu L, Lan X, Cohen D, Zhang Y, Ravindran AV, et al. (2019): Polyunsaturated fatty acids metabolism, purine metabolism and inosine as potential independent diagnostic biomarkers for major depressive disorder in children and adolescents. Mol Psychiatry 24: 1478-1488)

dGDP: deoxyguanosine diphosphate; dGTP: deoxyguanosine triphosphate.
