# Supplementary figures and images for: Chronic unpredictable mild stress produces depressive-like behavior, hypercortisolemia, and metabolic dysfunction in adolescent cynomolgus monkeys
Source: Transl Psychiatry. 2021 Jan 4;11:9. doi: 10.1038/s41398-020-01132-6 (PMC7791128; doi:10.1038/s41398-020-01132-6)

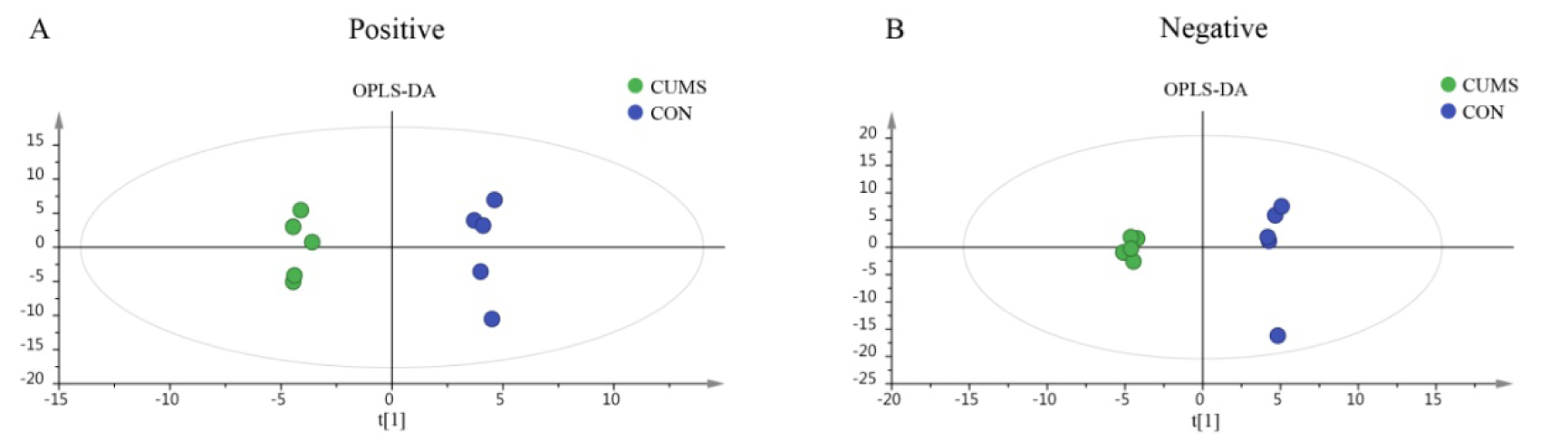

Supplement: Supplementary file 12 — Figure S1 [file 41398_2020_1132_MOESM12_ESM.tif]
